# Risk factors and prediction of distant metastasis (DM) of colon adenocarcinoma: a logistic regression and machine learning study based on surveillance, epidemiology, and end results (SEER) database

**DOI:** 10.1186/s12885-025-14329-z

**Published:** 2025-07-01

**Authors:** Qiang Guo, Junyun Li, Zhe Wei, Jingjing Xu, Shaojun Duan, Jianfeng Li, Yaxi Liu

**Affiliations:** 1 Team of Clinical Pharmacy, Department of Pharmacy, Jincheng People’s Hospital, Jincheng City, People’s Republic of China; 2 Department of General Surgery, Jincheng People’s Hospital, Jincheng City, People’s Republic of China; 3 Department of Information Technology, Jincheng People’s Hospital, Jincheng City, People’s Republic of China

**Keywords:** Colon adenocarcinoma, Distant metastasis, Risk factor, Machine learning

## Abstract

**Background:**

Given the limitations of traditional imaging examinations to detect distant metastasis (DM) (e.g., low sensitivity), this study is to identify pathological and laboratory risk factors and establish models predicting distant metastasis of colon adenocarcinoma (CA) patients.

**Methods:**

CA Patients diagnosed between the year of 2018 and 2021 were retrieved from SEER. Logistic regression was utilized to find independent risk factors (IRFs) of DM and 12 models including BNB (Bernoulli naïve bayes), DT (Decision tree), GBC (Gradient Boosting Classifier), GNB (Gaussian naïve bayes), KNN (K-nearest neighbor), LDA (Linear Discriminant Analysis), LR (Logistic regression), MLP (Multi-layer perceptron classifier), MNB (Multinomial naïve bayes), QDA (Quadratic discriminant analysis), RFC (Random forest classifier) and SVC (Support vector machine) were established and evaluated on the training set and test set (7:3) of the retrieved patients. Additionally, CA patient data was collected from Jincheng People’s Hospital (JCPH) as an external validation set for the prediction efficacy of the models.

**Results:**

7,000 and 83 CA patients were retrieved from SEER and JCPH respectively, and 8 IRFs including age 60–79 (OR = 0.589, 95% CI: 0.391–0.887) and age > 80 (OR = 0.456, 95% CI: 0.287–0.722), primary site – cecum (OR = 1.305, 95% CI: 1.023–1.664), TNM stage – T3 (OR = 8.869, 95% CI: 2.151–36.569) and T4 (OR = 15.912, 95% CI: 3.839–65.955), TNM stage – N1 (OR = 3.853, 95% CI: 2.919–5.087) and N2 (OR = 8.480, 95% CI: 6.322–11.374), number of regional nodes examined > 12 (OR = 0.439, 95% CI: 0.326–0.591), tumor deposits (OR = 1.989, 95% CI: 1.639–2.414), carcinoembryonic antigen (CEA) level (OR = 4.552, 95% CI: 3.747–5.530) and perineural invasion (OR = 1.352, 95% CI: 1.112–1.643) were identified. LR showed the best predictive efficacy both on the test (AUC = 0.892, sensitivity = 0.825, specificity = 0.801) and external validation set (AUC = 0.868, sensitivity = 1.000, specificity = 0.727).

**Conclusions:**

Machine learning is a promising way to assist the detection of DM for CA patients.

## Background

Colorectal cancer (CRC) is a common disease which ranks the 3rd and 2nd most commonly diagnosed cancer in males and females respectively. During these years, the incidence and mortality rates, which are significantly higher in males than females, have seen a slow but steady decreasing in the US, with approximately 153,000 new cases diagnosed annually, including 107,000 colon cancer cases, 46,000 rectal cancer cases, and more than 53,000 death cases [1–4].

Resection surgery, sometimes involving endoscopic resection for carefully chosen early-stage colon cancers, stands as the sole curative treatment option for localized colon cancer. Typically, patients diagnosed with distant metastasis (DM) are not suitable candidates for this surgery, except in cases where metastasis is limited, often to the liver or lungs. In such instances, there remains a possibility for curative resection through surgery. For patients who are no longer candidates for curative surgery, palliative surgery may still be an option to alleviate symptoms arising from the primary tumor, such as obstruction or bleeding [5]. This allowed us to explore the association between DM and pathological data after resection surgery.

Once a diagnosis of colorectal cancer (CRC) has been confirmed, disease staging becomes essential to inform subsequent therapeutic strategies and prognosis. The 8th edition (2017) of the Tumor, Node, Metastasis (TNM) staging system, jointly developed by the American Joint Committee on Cancer (AJCC) and the Union for International Cancer Control (UICC), remains the most widely endorsed staging framework.

Based on endoscopic, radiographic, and intraoperative findings, a clinical TNM stage (cTNM) can be determined. Alternatively, a pathological TNM stage (pTNM) can be established through histologic examination of the resected specimen after surgery. The key factor influencing survival outcomes following resection surgery is the pathological stage at the time of diagnosis, except in cases of rectal cancer patients undergoing neoadjuvant therapy, where the post-treatment pathologic stage serves as a more reliable prognostic indicator [6].

However, the TNM staging system solely takes into account the size of the primary tumor, lymph node involvement, and metastasis status, while disregarding the prognostic importance of other key factors like age, gender, differentiation grade, preoperative serum carcinoembryonic antigen (CEA) levels, lymphovascular invasion, and perineural invasion. Furthermore, it is universally recognized that the emergence of distant metastasis (DM) in cancer profoundly impacts the patient’s subsequent treatment plans and diminishes the prognosis. In the case of colon carcinoma, the onset of DM promptly categorizes the patient as stage IV (the terminal stage), associated with a 2-year relative survival rate of less than 40%, independent of the T/N stage [6]. At present, the main techniques for diagnosing DM include computed tomography (CT), positron emission tomography-CT (PET-CT), and various other imaging procedures. However, a significant hurdle persists: these methods often lack the sensitivity required to detect micro-metastases, poorly differentiated cells, or indolent cancer cells [7]. A study on peritoneal metastasis in CRC revealed that CT scans exhibited limited sensitivity, detecting only 11% of tumors smaller than 0.5 cm and 37% of those ranging from 0.5 to 5 cm [8]. Therefore, it is imperative to find alternative methods to assist the early detection of DM of CRC patients.

Currently, machine learning (ML) based on large-scale datasets shows promising potential in this field [7, 9]. And in this paper, we aimed to identify independent risk factors (IRFs) and build reliable models to assist the early detection of DM in colon adenocarcinoma (CA).

## Materials and methods

### Data source

Patient data were retrieved from “Incidence - SEER Research Data, 8 Registries, Nov 2023 Sub (1975–2021)” package of Surveillance, Epidemiology, and End Results (SEER) Database using the SEER*Stat 8.4.4 software. This package contains the data from 8 American cancer registries, covering about 8.3% of the US population [10]. We also collected the patient data (between 2020 and 2024) from the medical record system of Jincheng People’s Hospital (JCPH) as an external validation dataset.

The inclusion criteria were: (1) Patient age > 20 years; (2) Diagnosis between 2018 and 2021; (3) Site recode ICD-O-3/WHO 2008 = “Colon excluding Rectum”; (4) Primary Site - labeled = “C18.0 -Cecum”, “C18.2-Ascending colon”, “C18.3-Hepatic flexure of colon”, “C18.4-Transverse colon”, “C18.5-Splenic flexure of colon”, “C18.6-Descending colon” and “C18.7-Sigmoid colon”; (5) Histologically confirmed diagnosis (Diagnostic Confirmation = “Positive histology”); (6) Adenocarcinoma (Histology recode - broad groupings = “8140–8389: adenomas and adenocarcinomas”); (7) No other malignant tumor (First malignant primary indicator = “Yes”, Sequence Number = “One primary only”). The exclusion criteria were as follows: T stage = Tis; survival time < 1 month (to avoid the possible effects of surgery itself); other variables were missing or unknown.

### Variable selection and preprocessing

Based on clinical experience and literatures, 15 variables were included in this study - age, gender, race, marital status, primary site, TNM (tumor, node and metastasis stage), pathological grade, tumor size, regional lymph node examined/positive, tumor deposit, CEA level, perineural invasion (lymphovascular invasion data was not available in SEER).

Patient age was classified into 20-year intervals: “0” (20–39), “1” (40–59), “2” (60–79) or “3” (> 80); Race was categorized into “0” (white) or “1” (others). Marriage was classified into “0” (married) or “1” (others); T stage was classified into “0” (T1), “1” (T2), “2” (T3) or “3” (T4a and T4b); N stage was classified into “0” (N0), “1” (N1, N1a, N1b, N1c) or “2” (N2a, N2b); Pathologic grade was classified into “1” (well differentiated), “2” (moderately differentiated), “3” (poorly differentiated) or “4” (undifferentiated); Tumor size was classified into “0” (≤ 40 mm) or “1” (> 40 mm) [9]; Regional lymph node examined was classified into “0” (≤ 12 nodes) or “1” (> 12 nodes); Regional lymph node positive was classified into “0” (no positive nodes found) or “1” (≥ 1 positive nodes found); Tumor deposit was classified into “0” (no tumor deposits found) or “1” (≥ 1 tumor deposits found); Pretreatment CEA level was classified into “0” (negative/normal, borderline) or “1” (positive/elevated); Perineural invasion was classified into “0” (not identified/not present) or “1” (identified/present).

Besides, during the model establishment, we investigated adenocarcinoma originated from 7 primary sites (i.e., cecum, ascending, hepatic flexure, transverse, splenic flexure, descending colon and sigmoid). But for the primary site variable, classification into a “1, 2, 3, etc.” doesn’t make any sense since these numbers are just nominal and do not possess any intrinsic ordinal meanings. Thus, we created 6 artificial binary variables (also called “dummy” variables) – “Cecum (0 = No/1 = Yes)”, “Ascending colon (0 = No/1 = Yes)”, “Hepatic flexure (0 = No/1 = Yes)”, “Transverse colon (0 = No/1 = Yes)”, “Splenic flexure (0 = No/1 = Yes)” and “Descending colon (0 = No/1 = Yes)” to replace the original “primary site” variable and if all these 6 variables were “0”, primary site would be sigmoid (reference) [11].

### Statistical methods

In this study, the eligible patient cases from SEER were randomly divided into training set (70%) and test set (30%) and all variables were discretized and presented by N (%). Mann-Whitney-U test, Chi-squared or Fisher’s exact test were conducted to compare the variables between the training and test-set, according to whether these variables were ordinal.

Uni- and multi-variate logistic regressions were then performed on the whole SEER data (training set + test set) to identify the independent risk factors (IRFs) for DM. The outcome was expressed by odds ratios (OR) and their 95% confidence intervals (95% CIs). Correlations and muti-collinearity between the IRFs were investigated by Spearman correlation and variance inflation factor (VIF) analysis. IRFs with strong correlations may be considered for exclusion in the following model establishment.

After that, with these IRFs on hand and dummy classifier as the baseline model, we evaluated 12 prediction models on the training set, including BNB (Bernoulli naïve bayes), DT (decision tree), GBC (Gradient Boosting Classifier), GNB (Gaussian naïve bayes), KNN (K-nearest neighbor), LDA (Linear Discriminant Analysis), LR (Logistic regression), MLP (Multi-layer Perceptron classifier), MNB (Multinomial naïve bayes), QDA (Quadratic Discriminant Analysis), RFC (Random forest classifier), SVC (Support vector machine). In order to get a reliable evaluation on these models, we adopted a cross-validation (cv = 5, stratified sampling) technique to calculate the means of AUCs (area under the curves) of receiver operating characteristic curves (ROCs) and the best one or ones were then selected to be the candidates for further optimization. Hyperparameter tuning was then conducted for the candidate models through GridSearch cross-validation (cv = 5). Finally, again, a cross_validation (cv = 5, stratified sampling) was utilized to figure out the means of cut-off thresholds for each model by maximizing the Youden Indexes [12].

AUC, sensitivity, specificity, Youden index, precision, accuracy and F1 score were adopted to evaluate the candidate model performance both on the training and test set. In addition, Shapley additive explanations (SHAP) were also used for visualization and interpretation of the predicting effects of IRFs on the candidate models.

In this study, logistic regression was conducted by the IBM SPSS (24.0) software. Spearman, VIF analyses and model establishment were processed through Python pandas (version 2.0.3), numpy (version 1.24.3), seaborn (version 0.12.2), matplotlib (version 3.7.1) and scikit-learn package (version 1.3.0) in Jupyter Notebook (version 7.2.2). All statistical tests were two-tailed, and a *p* < 0.05 indicated statistically significant.

## Results

**Fig. 1:**
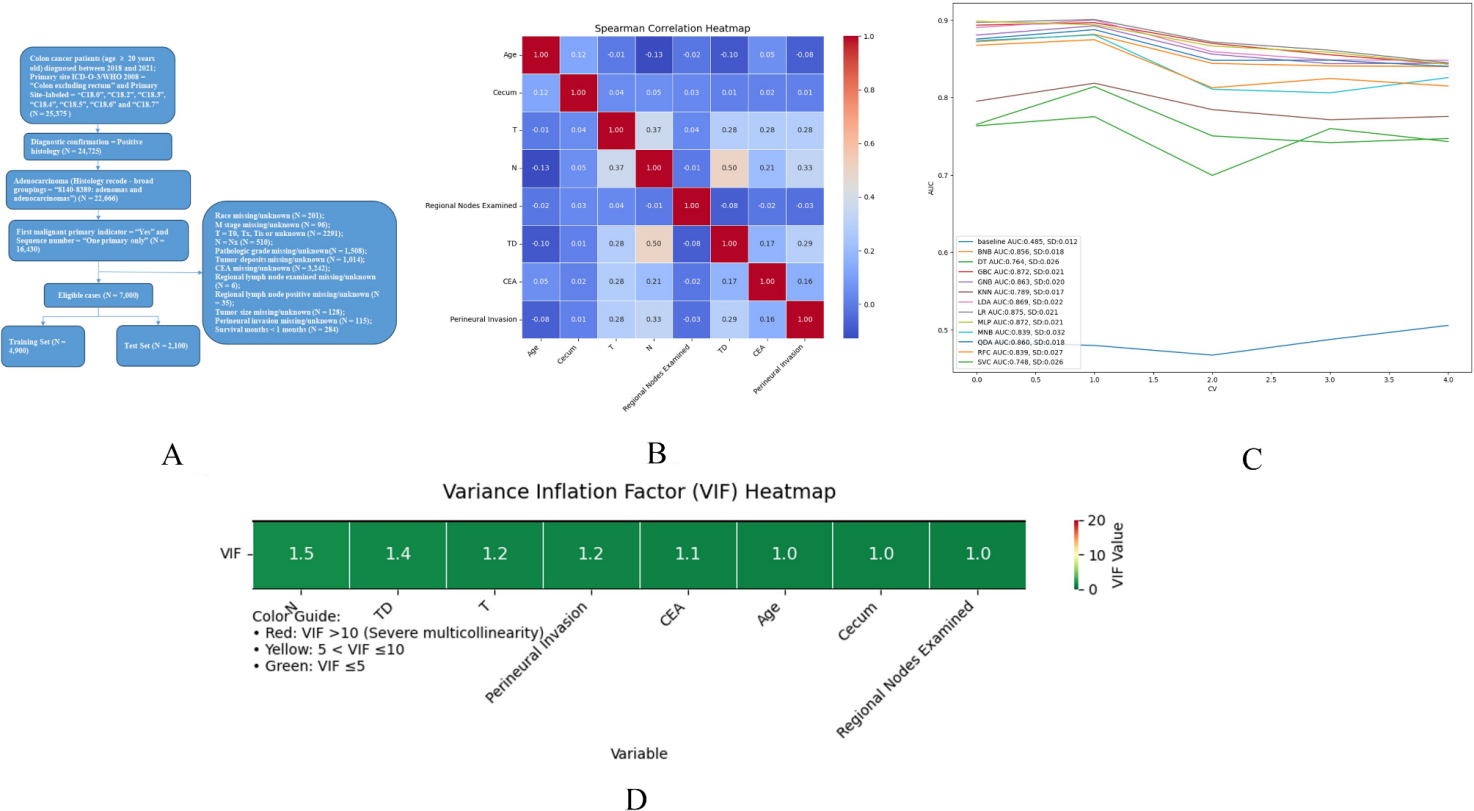
Flow chart of the Data Retrieve (**A**). Spearman Analysis of the IRFs (**B**). Cross_validation Evaluation of AUCs of the Models on the Training Set (**C**). Variance Inflation Factor Analysis of the IRFs (**D**). *SD = standard deviation

We got 25,375 cases which were diagnosed as colon cancer (rectal cancer excluded) between the year of 2018 and 2021 from SEER. Among these, there were totally 7,000 eligible cases meeting our inclusion and exclusion criteria (Figure 1A). The top 3 primary sites were “Sigmoid colon”, “Cecum” and “Ascending colon”. There were 789 cases (11.27%) who already had DM (i.e., their M stage was 1) when they were diagnosed. These 7,000 cases were then randomly divided into training (4,900 cases) and test sets (2,100 cases). Among the 15 variables, statistically significant differences existed (*p* < 0.05) for “Primary Site - cecum”, “T stage”, “Grade Pathological”, “Tumor Size Summary” and “CEA level” between the training and test set. We thought this kind of differences could be good for the validation of robustness of the following models we established, thus no further re-grouping operations were needed. 83 CA patients from JCPH were also collected and the demographic data of all the eligible cases were shown in Table 1.

**Table 1:** Demographic data of the eligible cases

Variables (*N*, %)	Total (*N* = 7,000)	Training Set (*N* = 4,900)	Test Set (*N* = 2,100)	*P* value	External Validation Set (*N* = 83)
Age				0.106	
20–39	243 (3.47%)	172 (3.51%)	71 (3.38%)		1 (1.20%)
40–59	2184 (31.20%)	1546 (31.55%)	638 (30.38%)		32 (38.55%)
60–79	3378 (48.26%)	2381 (48.59%)	997 (47.48%)		47 (56.63%)
> 80	1195 (17.07%)	801 (16.35%)	394 (18.76%)		3 (3.61%)
Race				0.183	
White	5155 (73.64%)	3631 (74.10%)	1524 (72.57%)		0 (0.00%)
Other (American Indian/AK Native, Asian/Pacific Islander)	1845 (26.36%)	1269 (25.90%)	576 (27.43%)		83 (100.00%)
Gender				0.137	
Male	3535 (50.50%)	2503 (51.08%)	1032 (49.14%)		39 (46.99%)
Female	3465 (49.50%)	2397 (48.92%)	1068 (50.86%)		44 (53.01%)
Marital Status				0.793	
Married	3830 (54.71%)	2676 (54.61%)	1154 (54.95%)		73 (87.95%)
Other	3170 (45.29%)	2224 (45.39%)	946 (45.05%)		10 (12.05%)
Primary Site				**0.001**	
Cecum	1563 (22.33%)	1121 (22.88%)	442 (21.05%)		4 (4.82%)
Ascending Colon	1518 (21.69%)	1031 (21.04%)	487 (23.19%)		31 (37.35%)
Hepatic Flexure	332 (4.74%)	211 (4.31%)	121 (5.76%)		2 (2.41%)
Transverse Colon	717 (10.24%)	527 (10.76%)	190 (9.05%)		1 (1.20%)
Splenic Flexure	272 (3.89%)	202 (4.12%)	70 (3.33%)		1 (1.20%)
Descending Colon	442 (6.31%)	327 (6.67%)	115 (5.48%)		13 (15.66%)
Sigmoid Colon	2156 (30.80%)	1481 (30.22%)	675 (32.14%)		31 (37.35%)
T Stage				**0.004**	
1	663 (9.47%)	436 (8.90%)	227 (10.81%)		2 (2.41%)
2	951 (13.59%)	651 (13.29%)	300 (14.29%)		5 (6.02%)
3	3834 (54.77%)	2698 (55.06%)	1136 (54.10%)		56 (67.47%)
4	1552 (22.17%)	1115 (22.76%)	437 (20.81%)		20 (24.10%)
N Stage					
0	3788 (54.11%)	2646 (54.00%)	1142 (54.38%)	0.717	47 (56.63%)
1	2082 (29.74%)	1489 (30.39%)	593 (28.24%)		24 (28.92%)
2	1130 (16.14%)	765 (15.61%)	365 (17.38%)		12 (14.46%)
M Stage				0.259	
0	6211 (88.73%)	4334 (88.45%)	1877 (89.38%)		77 (92.77%)
1	789 (11.27%)	566 (11.55%)	223 (10.62%)		6 (7.23%)
Grade Pathological (2018+)				**0.002**	
well differenciated	582 (8.31%)	354 (7.22%)	228 (10.86%)		24 (28.92%)
moderately differentiated	5332 (76.17%)	3781 (77.16%)	1551 (73.86%)		45 (54.22%)
poorly differentiated	1062 (15.17%)	745 (15.20%)	317 (15.10%)		10 (12.05%)
undifferentiated	24 (0.34%)	20 (0.41%)	4 (0.19%)		4 (4.82%)
Tumor Size Summary (2016+)				**0.042**	
<=40 mm	2988 (42.69%)	2053 (41.90%)	935 (44.52%)		23 (27.71%)
> 40 mm	4012 (57.31%)	2847 (58.10%)	1165 (55.48%)		60 (72.29%)
Regional nodes examined (1988+)				0.248	
<=12	529 (7.56%)	382 (7.80%)	147 (7.00%)		15 (18.07%)
> 12	6471 (92.44%)	4518 (92.20%)	1953 (93.00%)		68 (81.93%)
Regional nodes positive (1988+)				0.591	
0	4004 (57.20%)	2813 (57.41%)	1191 (56.71%)		23 (27.71%)
> 0	2996 (42.80%)	2087 (42.59%)	909 (43.29%)		60 (72.29%)
Tumor Deposits Recode (2010+)				0.353	
0	5829 (83.27%)	4067 (83.00%)	1762 (83.90%)		81 (97.59%)
> 0	1171 (16.73%)	833 (17.00%)	338 (16.10%)		2 (2.41%)
CEA Level				**0.022**	
negative/borderline	4284 (61.20%)	2956 (60.33%)	1328 (63.24%)		49 (59.04%)
positive	2716 (38.80%)	1944 (39.67%)	772 (36.76%)		34 (40.96%)
Perineural Invasion				0.099	
negative	5852 (83.60%)	4073 (83.12%)	1779 (84.71%)		44 (53.01%)
positive	1148 (16.40%)	827 (16.88%)	321 (15.29%)		39 (46.99%)

### Independent risk factors (IRFs)

After uni- and multi-variate logistic regression analyses, 8 variables - age, primary site (only the cecum subgroup), T stage, N stage, regional lymph node examined, tumor deposits, CEA level and perineural invasion were identified as IRFs of distant metastasis (DM). 60–79 years (OR = 0.589, *p* = 0.011) and > 80 years (OR = 0.456, *p* = 0.001) of age and over 12 regional lymph nodes examined (OR = 0.439, *p* < 0.001) were negatively associated with DM and the others were positively associated (Table 2). No significant correlation or multi-collinearity was observed among the IRFs in the Spearman and VIF analysis (Figure 1B/D).

**Table 2:** Logistic regression of distant metastasis of eligible cases

Variables	Univariate		Multivariate		Overall P-value (Multivariate)
	OR (95% CI)	P-value	OR (95% CI)	P-value	
Age					< 0.001
20–39 (reference)					
40–59	0.724 (0.514, 1.020)	0.065	0.842 (0.559, 1.270)	0.413	
60–79	0.467 (0.332, 0.656)	< 0.001	0.589 (0.391, 0.887)	0.011	
> 80	0.37 (0.252, 0.543)	< 0.001	0.456 (0.287, 0.722)	0.001	
Race					0.366
White (reference)					
Other (American Indian/AK Native, Asian/Pacific Islander)	1.28 (1.090, 1.505)	0.003	1.093 (0.901, 1.327)	0.366	
Gender					
Male (reference)					
Female	1.118 (0.964, 1.296)	0.142			
Marital Status					0.384
Married (reference)					
Other	1.22 (1.052, 1.415)	0.009	1.082 (0.906, 1.291)	0.384	
Primary Site					0.148
Cecum	1.23 (1.013, 1.494)	0.037	1.305 (1.023, 1.664)	0.032	
Ascending colon cancer	0.711 (0.569, 0.888)	0.003	1.086 (0.827, 1.425)	0.553	
Hepatic Flexure	0.591 (0.383, 0.914)	0.018	0.783 (0.473, 1.297)	0.342	
Transverse Colon	0.953 (0.730, 1.245)	0.725	1.309 (0.952, 1.800)	0.097	
Splenic Flexure	1.347 (0.942, 1.926)	0.102	1.263 (0.824, 1.937)	0.284	
Descending Colon	0.86 (0.615, 1.203)	0.379	0.921 (0.626, 1.353)	0.674	
Sigmoid Colon (reference)					
T Stage					< 0.001
1 (reference)					
2	5.656 (1.296, 24.680)	0.021	4.183 (0.938, 18.647)	0.061	
3	34.669 (8.616, 139.497)	< 0.001	8.869 (2.151, 36.569)	0.003	
4	117.479 (29.185, 472.888)	< 0.001	15.912 (3.839, 65.955)	< 0.001	
N Stage					< 0.001
0 (reference)					
1	8.338 (6.474, 10.740)	< 0.001	3.853 (2.919, 5.087)	< 0.001	
2	25.33 (19.645, 32.659)	< 0.001	8.48 (6.322, 11.374)	< 0.001	
Grade Pathological (2018+)					0.627
well differentiated (reference)					
moderately differentiated	2.257 (1.529, 3.333)	< 0.001	1.279 (0.822, 1.992)	0.275	
poorly differentiated	4.906 (3.261, 7.381)	< 0.001	1.35 (0.841, 2.168)	0.214	
undifferentiated	3.957 (1.267, 12.357)	0.018	1.707 (0.463, 6.291)	0.422	
Tumor Size Summary (2016+)					0.103
<=40 mm (reference)					
> 40 mm	2.094 (1.778, 2.466)	< 0.001	1.178 (0.967, 1.433)	0.103	
Regional nodes examined (1988+)					< 0.001
<=12 (reference)					
> 12	0.49 (0.390, 0.617)	< 0.001	0.439 (0.326, 0.591)	< 0.001	
Regional nodes positive (1988+)					0.203
0 (reference)					
> 0	9.208 (7.536, 11.251)	< 0.001	1.305 (0.866, 1.965)	0.203	
Tumor Deposits Recode (2010+)					< 0.001
0 (reference)					
> 0	7.546 (6.438, 8.845)	< 0.001	1.989 (1.639, 2.414)	< 0.001	
CEA Level					< 0.001
negative/borderline (reference)					
positive	6.97 (5.842, 8.317)	< 0.001	4.552 (3.747, 5.530)	< 0.001	
Perineural Invasion					0.003
negative (reference)					
positive	4.495 (3.831, 5.274)	< 0.001	1.352 (1.112, 1.643)	0.003	

Notes: Bold font means P-value < 0.05

### Establishment of the prediction models

On the basis of the 8 IRFs, 12 machine learning classifiers were established and evaluated for DM in the CA patients on the data of training set (N = 4,900). Figure 1C presented the cross-validation of AUCs of the models. We could see that LR (means of AUCs: 0.875), GBC (means of AUCs: 0.872), and MLP (means of AUCs: 0.872) made the top 3 performances on AUC. After hyperparameter tuning, the best hyperparameters were set respectively for LR (C = 0.1, class_weight = None, penalty = ‘l1’, solver = ‘saga’), GBC (learning_rate = 0.1, max_depth = 3, max_features = ‘sqrt’, min_samples_leaf = 2, min_samples_split = 2, n_estimators = 50, subsample = 0.9) and MLP (activation = ‘relu’, alpha = 0.001, hidden_layer_sizes = (100,), learning_rate_init = 0.001, max_iter = 400, solver= ‘adam’). A cross validation (cv = 5) was utilized on the training set again to figure out the cut-off thresholds for LR (0.121), GBC (0.120) and MLP (0.138).

The performance metrics, including AUC, sensitivity, specificity, Youden Index, precision, accuracy, and F1 score, were computed for the three models on both the training and test datasets. Among these, Logistic Regression (LR) stood out with the highest performance on the test set, achieving an AUC of 0.892, sensitivity of 0.825, and specificity of 0.801. To confirm its reliability, LR’s performance was further assessed on an external validation set, where it maintained a strong showing with an AUC of 0.868, perfect sensitivity of 1.000, and specificity of 0.727 (Table 3).

**Table 3:** Evaluation of the LR, GBC and MLP models on the training, test and external validation dataset

Dataset	Models	AUC	Sensitivity	Specificity	Youden Index	Precision	Accuracy	F1 Score
Training Set	LR	0.878	0.829	0.782	0.615	0.332	0.787	0.474
	GBC	0.883	0.813	0.803	0.619	0.350	0.804	0.490
	MLP	0.885	0.781	0.835	0.619	0.382	0.829	0.513
Test Set	LR	0.892	0.825	0.801	0.626	0.330	0.804	0.472
	GBC	0.889	0.807	0.799	0.606	0.323	0.800	0.462
	MLP	0.889	0.713	0.861	0.574	0.379	0.845	0.495
	LR	0.868	1.000	0.727	0.727	0.222	0.747	0.364
External Validation Set	GBC	0.848	0.833	0.740	0.574	0.200	0.747	0.323
	MLP	0.840	0.833	0.740	0.574	0.200	0.747	0.323

**Fig. 2:**
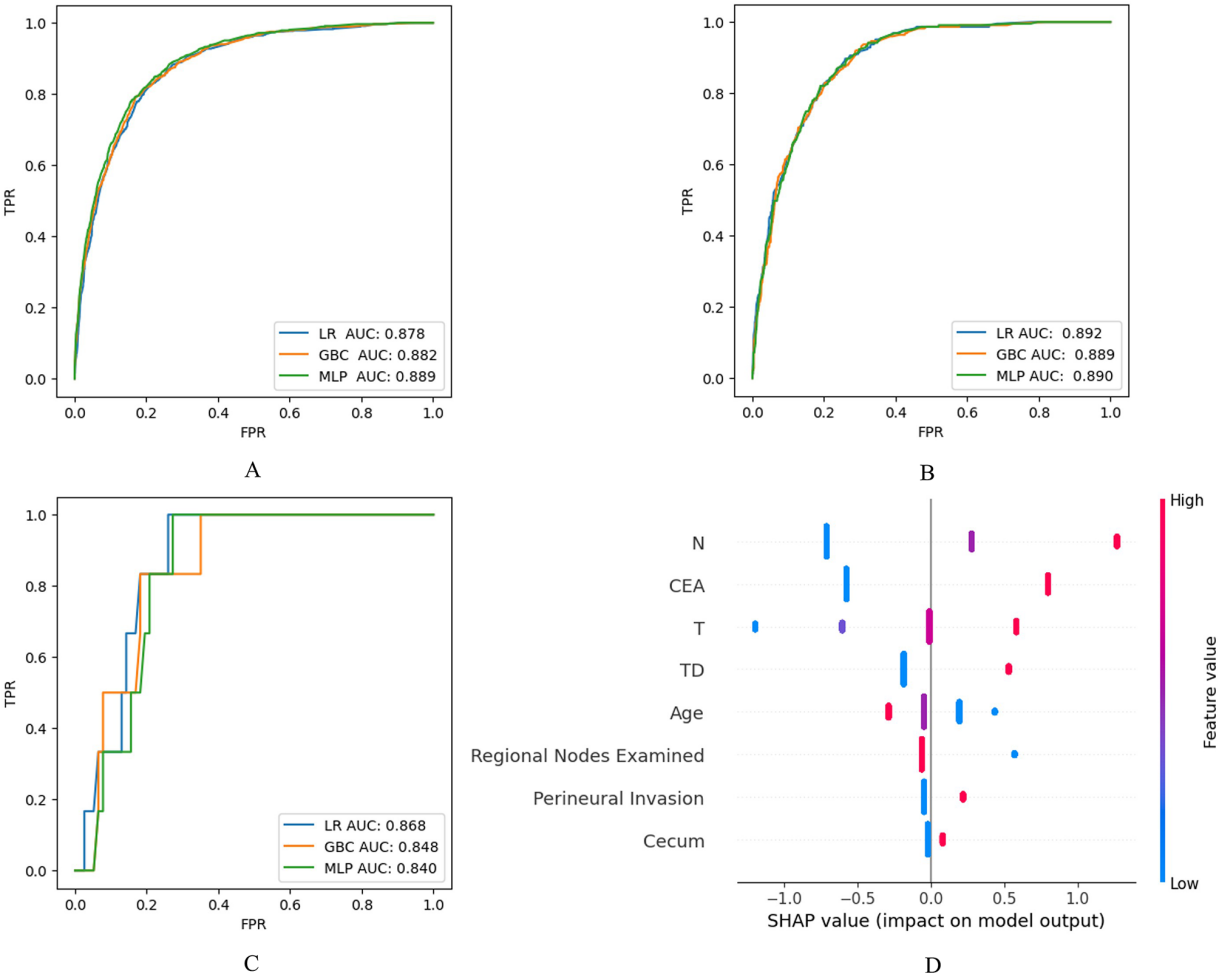
ROCs of the LR, GBC and MLP on training set (**A**), test set (**B**) and external validation set (**C**). SHAP Plot for the LR on Test Set (**D**): Each point on the summary plot is the Shapley value for one IRF and one instance. All IRFs are in descending order of importance along the vertical axis (from top to bottom). The colors represent the values of the IRFs from low (blue) to high (red). The point on the right of the vertical axis (i.e., positive Shapley value) means the IRF increase the possibility of predicting M1

The predictive effects of IRFs in the LR model on the test set were visualized in the SHAP summary plot for clearer interpretation (Figure 2D). This plot reveals the relative importance of the eight IRFs: in descending order, they are N stage, CEA level, T stage, tumor deposits, age, number of regional lymph nodes examined, perineural invasion, and cecum.

## Discussion

Colorectal cancer is a common malignant tumor originating from colon and rectum and adenocarcinoma accounts for the vast majority of histological type (> 90%), while other types, including neuroendocrine neoplasms, hamartomas, mesenchymal tumors, lymphomas are unusual. Pathological analysis of the resected specimen after resection surgery is the most powerful tool for assessing prognosis of these patients and the TNM staging system is commonly used to determine the disease stage [6]. While the T/N stage can be confirmed through pathological examination, the assessment of the M stage has, to date, predominantly depended on conventional imaging techniques, whose limited sensitivity poses a challenge for the early identification of distant metastases.

Once DM occurs, the prognosis of the patients would be extremely poor. While the pathological M1 stage can be assigned when DM is confirmed by imaging and pathological assessment, verifying pathological M0 is nearly impossible, since that would denote the absence of DM anywhere in the body which is nearly impossible to be validated [6]. Thus, the M0 stage of TNM staging system is actually only a clinical estimation based on traditional imaging examinations and it is imperative to find another way to do this.

In this study, we firstly identified 8 independent risk factors for DM through logistic regression - age, cecum, T stage, N stage, regional nodes examined, TD, CEA and perineural invasion.

Age, specifically between 60 and 79 years (OR = 0.589, 95% CI = 0.391, 0.887) and over 80 years (OR = 0.456, 95% CI = 0.287, 0.722), was negatively associated with DM risk. This aligns with observations indicating that early-onset colorectal cancer patients (those under 50 years of age) tend to exhibit a more aggressive disease course and increased metastasis risk compared to those with late-onset disease (over 50 years) [13].

Regarding the primary site, with sigmoid as the reference, only cecum showed a statistically higher risk for DM (OR = 1.305, 95% CI = 1.023, 1.664). The prognosis of cecum cancer is somewhat poorer than other types of colon cancer, most likely due to the great difficulties in early diagnoses [14]. Numerous studies have demonstrated that the location of the primary tumor serves as a prognostic factor in colorectal cancer (CRC). A comprehensive meta-analysis, encompassing 1,427,846 patients across all stages of CRC, revealed that a left-sided tumor location is significantly associated with a reduced risk of death (OR = 0.820, 95% CI = 0.79, 0.84) [15–17]. However, Ramzi Amri et al. found right-sided colon cancer is less likely to spread to liver or lung than left-sided one [18]. Frankly speaking, the risk of death does not equate to the risk of DM, yet it is widely agreed that the existence of DM serves as a potent predictor for cancer patients’ survival. Our study findings suggest a potential need to further investigate the association between cecum cancer and its corresponding DM risk.

TNM staging system is a widely favored tool to stratify CRC patients. In this study, the significant association between T_3_ (OR = 8.869, 95% CI = 2.151, 36.569)/T_4_ (OR = 15.912, 95% CI = 3.839, 65.955), N_1_ (OR = 3.853, 95% CI = 2.919, 5.087)/N_2_ (OR = 8.480, 95% CI = 6.322, 11.374) and DM aligns with the stratification of the staging system that advanced T/N stages imply poorer prognosis [19].

The number of regional nodes examined > 12 (OR = 0.439, 95% CI = 0.326, 0.591) was observed as a protective factor for DM of CA in this study. Other studies have also found the number of regional lymph nodes collected in a resection surgery directly influences prognosis for stage II (node – negative) and stage III (node - positive) colon cancer [20–22]. The reason for the relationship between the prognosis and total number of the examined lymph nodes is not clear yet. The larger number of examined nodes may be a result of a more thorough resection surgery. Another hypothesis is that the tumor factors from immune system (a reflection of immunity recognition) could stimulate lymph nodes to enlarge, which make the nodes easier to be recognized and counted [6].

In the eighth edition of AJCC TNM staging, tumor deposits (TDs) are defined as “discrete tumor nodules within the lymph drainage area of the primary carcinoma without identifiable lymph node tissue or identifiable vascular or neural structure”. They are considered the equivalent of nodal metastases and if positive, even in the absence of any identified regional lymph nodes, patients would directly enter stage III. In our study, TD was identified as an IRF for DM (OR = 1.989, 95% CI = 1.639, 2.414), which was also consistent with its adverse effect on prognosis.

High level of serum preoperative serum carcinoembryonic antigen (CEA) is considered an important prognosis factor in colon cancer. Some studies found CEA level ≥ 5.0 ng/mL poses a negative impact on survival regardless of tumor stage but the optimal cutoff value is still debated [23–26]. In this paper, CEA level was categorized into binary classes (i.e., within or above the normal range) and still showed a significant association with DM (OR = 4.552, 95% CI = 3.747, 5.530).

There are robust evidences supporting that the existence of perineural invasion (PNI) is associated with poor survival so that official guidelines recommend PNI to be included in the definition of “high-risk” stage II colon cancer and might affect the adjuvant chemotherapy plans in this setting [27–31]. We also observed a slightly higher risk of DM for PNI (OR = 1.352, 95% CI = 1.112, 1.643).

Besides, we found pathological grade, tumor size were not IRFs for DM. Pathological grade, although, reflects the degree of the tumor differentiation and has been demonstrated to be an independent prognostic factor, the subjectiveness and significant variability of the grading process undermines its reliability [6]. It is widely believed that tumor size does not significantly affect the prognosis of colorectal cancer [32, 33]. Although, there is a relatively new study suggesting that tumor size (especially > 4.5 cm) may be a significant predictor of poor outcome for colon, not rectal, cancer [34].

12 models were established and evaluated for DM prediction. Among them, LR showed the best prediction efficacies on the test and external validation set. SHAP also gave an interpretation on the influence of IRFs on the LR model in predicting DM for the test set. Basically, it aligned with the logistic regression analysis above.

The model offered another possible way to evaluate the DM risk of CA patients after resection surgery, especially those without obvious DM after traditional imaging examinations - if a CA patient got a predicted M1, the doctor may need to consider a more intense follow-up and even a more aggressive therapy. For instance, biologic agents (e.g., bevacizumab, cetuximab/panitumumab) are usually recommended to be added to conventional chemotherapy in CA patients already with DM (i.e., stage IV) but not in the stage II – III patients [35]. A large clinical trial found a disappointing result that the addition of cetuximab to the modified sixth version of the FOLFOX regimen (mFOLFOX6) showed no obvious benefits in the treatment of stage III colon cancer [36]. However, the study did not consider a further stratification of the stage III patients and according to our study, what if a new trial were to categorize stage III patients into predicted M0 and predicted M1 subgroups? Could the predicted M1 cohort potentially reap the benefits of these medications?

Above all, we believe that this LR model holds significant promise as a complement to traditional imaging examinations for early detection of DM in CA patients. However, our study has limitations, and further research could refine our findings in several ways. Firstly, the external validation set had a relatively small and unbalanced sample size, with only 6 “M1” cases among 83 total, affecting the robustness of the validation and resulting in low precision rates (positive predictive values of 0.222 for the LR model). Secondly, the “Incidence - SEER Research Data, 8 Registries, Nov 2023 Sub (1975–2021)” package lacks data on molecular biomarkers such as microsatellite instability/mismatch repair and RAS/BRAF mutations, which are considered valuable in colorectal cancer prognosis. Their role in predicting DM needs evaluation, and their inclusion could potentially enhance the model’s effectiveness. Thirdly, as a preliminary study, this research only explores the potential of machine learning in detecting DM, leaving its clinical value, such as survival stratification for patients predicted as M0 or M1, unclear. More rigorous retrospective or prospective cohort studies are necessary to assess the model’s clinical significance. Lastly, our study population was predominantly white (73.64%), so our findings should be interpreted cautiously when applied to other ethnic groups.

## Conclusion

As we know, this is the first tentative study to identify risk factors, establish models predicting distant metastasis of CA patients on the basis of their demographic, pathological and laboratory information. As we could see, the models (especially the LR) showed quite satisfying prediction efficacy, which could be a new powerful assistant for DM detection in addition to traditional imaging examinations. Furthermore, the models may also offer a possible way to further stratify CA patients without DM into predicted M0/1, calling for additional survival studies and if possible, new clinical trial designs investigating the role of bioagents in stage III CA patients. Ultimately, machine learning emerges as a viable approach to enhance DM detection and refine the management of CA patients.

## Data Availability

Raw data used in the analyses is available in SEER database (http://seer.cancer.gov/seerstat). We could offer the associated python source code file (“Prediction Model.ipynb”) for LR, GBC and MLP to help our readers establish their own local models if needed.

## Abbreviations

AJCC: American Joint Committee on Cancer
AUC: Area under the curve
BNB: Bernoulli naïve bayes
CA: Colon adenocarcinoma
CEA: Carcinoembryonic antigen
CRC: Colorectal cancer
CT: Computed tomography
DM: Distant metastasis
DT: Decision tree
GBC: Gradient Boosting Classifier
GNB: Gaussian naïve bayes
IRFs: Independent risk factors
JCPH: Jincheng People’s Hospital
KNN: K-nearest neighbor
LDA: Linear Discriminant Analysis
LR: Logistic regression
ML: Machine learning
MLP: Multi-layer perceptron classifier
MNB: Multinomial naïve bayes
PET-CT: Positron emission tomography-CT
PNI: Perineural invasion
QDA: Quadratic Discriminant Analysis
RFC: Random forest classifier
ROC: Receiver operating characteristic curves
SEER: Surveillance, Epidemiology, and End Results Database
SHAP: Shapley additive explanations
SVC: Support vector machine
TD: Tumor deposit
TNM: Tumor, Node, Metastasis staging system
UICC: Union for International Cancer Control
VIF: Variance inflation factor
